# Next-generation cell line selection methodology leveraging data lakes, natural language generation and advanced data analytics

**DOI:** 10.3389/fbioe.2023.1160223

**Published:** 2023-06-05

**Authors:** Stephen Goldrick, Haneen Alosert, Clare Lovelady, Nicholas J. Bond, Tarik Senussi, Diane Hatton, John Klein, Matthew Cheeks, Richard Turner, James Savery, Suzanne S. Farid

**Affiliations:** ^1^ Department of Biochemical Engineering, University College London, London, United Kingdom; ^2^ Cell Culture and Fermentation Science, Biopharmaceuticals Development, R&D, AstraZeneca, Cambridge, United Kingdom; ^3^ Analytical Sciences, Biopharmaceuticals Development, R&D, AstraZeneca, Cambridge, United Kingdom; ^4^ Data Science and Modelling, Biopharmaceuticals Development, R&D, AstraZeneca, Cambridge, United Kingdom; ^5^ Purification Process Sciences, Biopharmaceuticals Development, R&D, AstraZeneca, Cambridge, United Kingdom

**Keywords:** cell line development, machine learning, data analytics, natural language generation, Industry 4.0

## Abstract

Cell line development is an essential stage in biopharmaceutical development that often lies on the critical path. Failure to fully characterise the lead clone during initial screening can lead to lengthy project delays during scale-up, which can potentially compromise commercial manufacturing success. In this study, we propose a novel cell line development methodology, referenced as *CLD*_*4*_, which involves four steps enabling autonomous data-driven selection of the lead clone. The first step involves the digitalisation of the process and storage of all available information within a structured data lake. The second step calculates a new metric referenced as the cell line manufacturability index (*MI*_*CL*_) quantifying the performance of each clone by considering the selection criteria relevant to productivity, growth and product quality. The third step implements machine learning (ML) to identify any potential risks associated with process operation and relevant critical quality attributes (CQAs). The final step of *CLD*_*4*_ takes into account the available metadata and summaries all relevant statistics generated in steps 1–3 in an automated report utilising a natural language generation (NLG) algorithm. The *CLD*_*4*_ methodology was implemented to select the lead clone of a recombinant Chinese hamster ovary (CHO) cell line producing high levels of an antibody-peptide fusion with a known product quality issue related to end-point trisulfide bond (TSB) concentration. *CLD*_*4*_ identified sub-optimal process conditions leading to increased levels of trisulfide bond that would not be identified through conventional cell line development methodologies. *CLD*_*4*_ embodies the core principles of Industry 4.0 and demonstrates the benefits of increased digitalisation, data lake integration, predictive analytics and autonomous report generation to enable more informed decision making.

## 1 Introduction

Cell line development (CLD) is a critical task within biopharmaceutical manufacturing and selects the lead clone for the master cell bank (MCB) which provides the starting material for the entire life span of a therapeutic drug candidate. Traditional methods for CLD are time-consuming and require a large number of experiments to identify the best candidate, which can lead to delays in the development timeline and higher costs. This selection process typically aims to achieve two objectives. The first is to reduce a large heterogeneous pool of between a 1,000–10,000 cell lines to a single clone expressing high levels of a therapeutic protein that meet all relevant product quality specifications (Munro et al., 2017). The second is to ensure the selected lead clone will scale-up appropriately and consistently deliver the required product demand whilst achieving the desired product quality specifications. Both objectives are equally important; however, the majority of research focuses on the first objective of selecting a high producing clone, with scale-up considerations a secondary objective. This oversight is normally due to strict timelines and availability of data resulting in a decision on the lead clone selection before an in-depth evaluation of all scale-up and process considerations can be properly accessed. This paper proposes a more holistic methodology for CLD that better leverages the data generated during CLD to select the lead clone to satisfy productivity, quality and scalability objectives.

**FIGURE 1:**
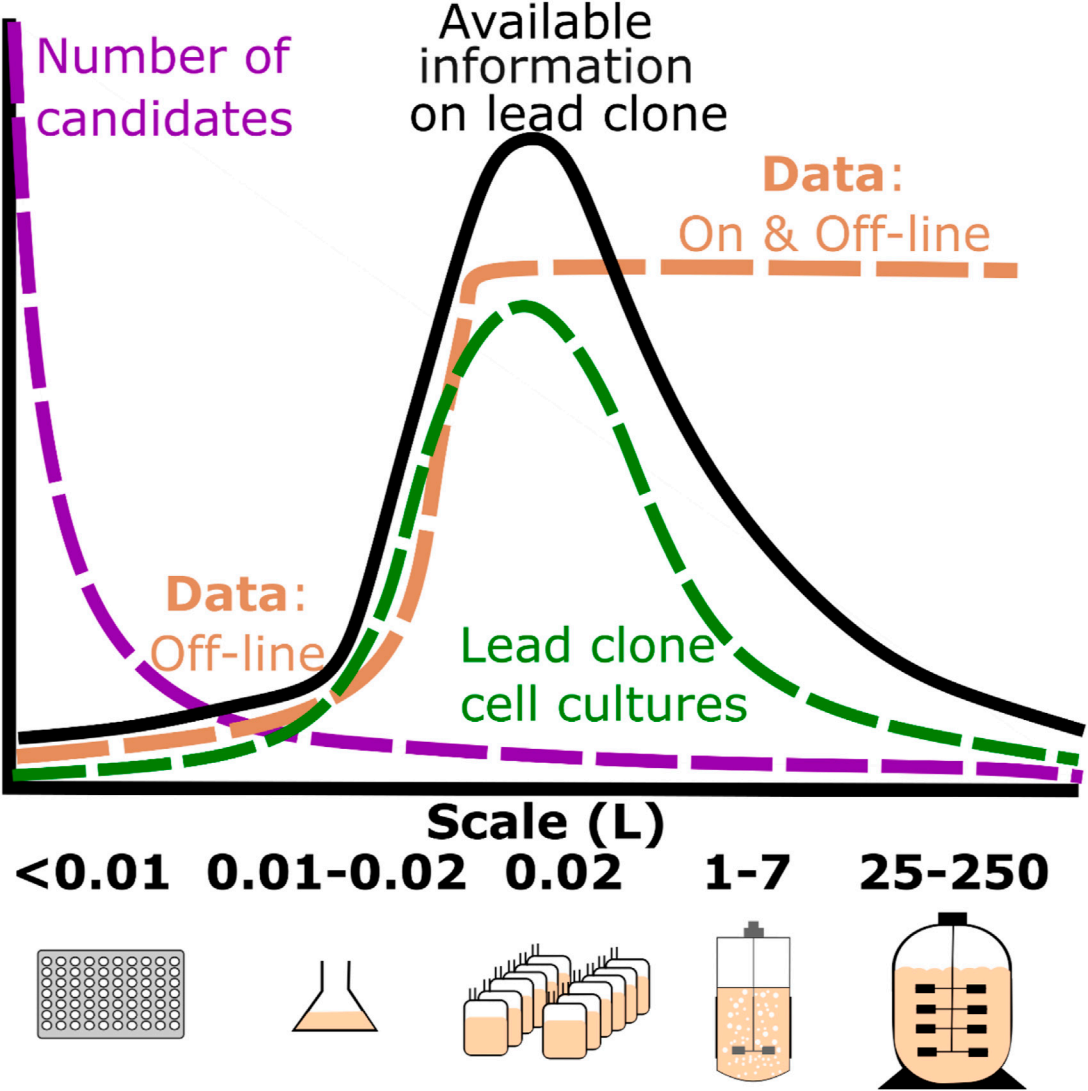
Schematic diagram summarizing the number of candidate clones, lead clone cell cultures, data recorded and available information on the lead clone for a typical therapeutic protein as it transitions from micro-scale (<0.1 L) to pilot scale manufacturing (250 L). The number of clone candidates is highlighted by the purple line, the number of lead clone culture runs is shown by the green line and data available at each scale by the brown line which is primarily defined by either off-line analytics or both off-line and online process data recorded at each scale. The available information trend line shown in black is a function of the number of lead clone cultures multiplied by the data available at the various scales.

A summary of some CLD related challenges is depicted in Figure 1. The first challenge relates to the early stages of CLD where the focus is on high-throughput evaluation of thousands of clone candidates cultured using a low volume (<1 mL) and operated in batch mode. The initial screening typically involves a high number of candidates assessed using high-throughput 96 well plates or microtitre plates (MTPs). Subsequent screening of hundreds of clones can be performed in high throughput fed-batch using multi-well plates in automated culturing systems (Rameez et al., 2014; Silk et al., 2010). The early stages of CLD follow a well-established procedure to appropriately select the cell lines that can move forward (Lin et al., 2019; Li et al., 2010; Hong et al., 2018). The cell lines are transfected with the gene of interest for the targeted product, methods include transfecting plasmids through calcium phosphate, cationic lipid-based lipofection, electroporation and using polymer-based reagents amongst others (Li et al., 2010). These high-throughput platforms screen using cell markers, typically MTX (methotrexate) or MSX (methionine sulfoximine) for glutamine synthetase (GS) mediated cells (Li et al., 2010; Lin et al., 2019). Each selected cell is then cloned into its own colony, as regulators require evidence that the cells are derived from a single progenitor cell to ensure monoclonality (European Medicines Agency, 2006; Chen et al., 2020). Methods such as single cell printing using the ClonePix system and Fluorescence-activated cell sorting (FACS) flow cytometry are methods implemented to ensure separate cell lines for their colonies (Kim, Diao, and Shuler, 2012). In order to identify a stable highly expressing cell line that meets the desired quality requirements a high number of candidates are required to be assessed. This involves various analytical assays such as ELISA to eliminate non-producing cells, followed by subsequent screening for highly productive clones (Kim, Diao, and Shuler, 2012; Frye et al., 2016). These initial cell line screenings experiments typically involve the use of high-throughput 96 well plates or microtitre plates (MTPs). These experiments are time-consuming, do not accurately reflect scale-up conditions and require a significant amount of analytical equipment to evaluate best performing cell lines. Furthermore, the low volume of these batch cultures during initial screening restricts the availability of off-line analytics resulting in the assessment criteria for each clone solely dependent on protein concentration and/or cell growth metrics.

In order to speed up the selection of the most productive cell lines and reduce product development timelines, the industry has developed different high throughput (HT) methods. Systems such as microtitre plates (MTPs) with a volume of 100–400 μL are great during initial screening allowing for HT runs in parallel, however they lack automation and their orbital shaking design can generate variability in comparison to other systems (Li et al., 2010; Huang and Kwiatkowski, 2015). Deep well plates (DWP) with a volume of 0.5–2 mL also have HT capabilities however similar to MTPs can be affected by poor mixing as their shaking is not comparable to mixing performed by impellers (Ben-Tchavtchavadze, Perrier, and Jolicoeur, 2007; Huang and Kwiatkowski, 2015). Additional automated systems have also been developed such as the SimCell™ microfluidic and micro-bioreactor systems; the Pall Micro24 system with a deep well design, each mini reactor has a standard baffled design and a working volume of up to 7 mL (Warr et al., 2013; Huang and Kwiatkowski, 2015). These systems have control units that monitor cell culture process parameters and can successfully predict key product quality attributes; it has also shown potential for scale-up applications through media screening and process optimisation (Ravindran et al., 2019; Huang and Kwiatkowski, 2015; Whitford, 2006). Other popular HT microbioreactor systems include the ambr^®^ 15 cell culture and ambr^®^ 250 systems that contain impellers and are comparable scale-down mimics of bench-scale systems (Huang and Kwiatkowski, 2015).

As shown in Figure 1, the micro-bioreactor stage is one of the most important stages as it is the first stage where both off-line and on-line process variables are recorded. At this stage the number of clones has been reduced to manageable numbers in the region of 10–100 clones. Additionally, this is the first stage of the selection process that evaluates the impact of controlling the key environment variables (e.g., pH and dissolved oxygen) in conjunction with fed-batch operation on the key productivity and growth metrics of each clone. This stage of the CLD process is a critical decision point in the selection of the lead clone as all the necessary information is available to make an informed decision on the optimum choice of lead clone as both process and off-line data analytics are available. However, this decision is heavily focused on off-line productivity metrics such as final titre with little consideration of the on-line variables such as pH or dissolved oxygen (DO_2_). Yet, these environmental variables can have a major influence on the metabolism of mammalian cell cultures; for example, Konakovsky et al. (2016) demonstrated the impact of adjusting the pH set-point on the production and consumption rates of lactic acid in addition to influencing the glucose consumption rates. Therefore, it is important to better understand the impact of these environment variables on the available productivity and growth metrics at this stage as they may play a significant role as the lead clone is scaled to commercial manufacturing. The on-line data is therefore a very valuable asset as it can help assess problems during scale-up such as evaluating the oxygen consumption rate of the cells to ensure the final commercial-scale bioreactor can meet the oxygen requirements for those high yielding cells (Garcia-Ochoa and Gomez, 2009). Furthermore, having access and analysing all of this data might highlight some latent operation issues that require subsequent evaluation and could pose a material impact on the tight timelines.

This paper outlines the development of a data-driven methodology to enhance lead clone selection that not only considers the available titre concentration and product quality that is the primary source of information during conventional CLD but also leverages the significant untapped data resource containing all the available off-line, on-line and metadata. To help automate this selection process this paper outlines the use of a simple natural language generation (NLG) algorithm to evaluate all available information which summarises and contextualises the large volume of information into a human readable report. This automatically generated report outlines the key metrics and other useful correlations to assist the operator for their lead clone selection. This methodology aims to remove a significant burden of time on all scientists and engineers that repeatedly spend large amounts of time on writing up and presenting results on this critical business decision. This autonomous cell line selection protocol demonstrates a novel application of NLG within the biopharmaceutical space that leverages all data within the data lake. This enables more informed data-driven decisions that pave the way towards Industry 4.0 implementation within the bioprocessing manufacturing sector.

## 2 Materials and methods

### 2.1 Cell line, culture propagation, and bioreactor systems

All cell lines used a Chinese hamster ovary (CHO) host that expressed high levels of an antibody-peptide fusion protein and were cultivated in chemically defined CHO media. The cells were maintained at 37°C under 5% carbon dioxide, shaken at a constant rpm and passaged 2–3 times per week for propagation and scale-up for inoculation. The cell line screening was carried out on an advanced micro-bioreactor ambr^®^ 15 system (Sartorius., 2020) with 48 single vessels split into four separate culture stations where each vessel was operated with a 11–15 mL working volume. The temperature and pH of each culture station was controlled to 33°C and 7.0, respectively and the agitation rate was ramped up to ensure the dissolved oxygen concentration of 50% could be maintained. The feeding strategy involved five equally spaced additions of the feed after the initial feed day indicated. The culture pH was controlled to 7.0 through the addition of sodium carbonate and sparging with CO_2_ gas with its control strategy implementing a pH dead-band equal to 0.1. Antifoam was added as required. Daily at-line samples were analysed for viable cell density (VCD) and viability (Viab) using the Vi-Cell Automated Cell Viability Analyzer (Beckman Coulter, Brea, CA, United States), and glucose (Gluc) and lactate (Lact) were analysed using the 2900D Biochemistry Analyzer (YSI, Yellow Springs, Ohio, United States). The rpm set-points, initial seeding density, and supplemented feeds contain proprietary information, which prevents us from providing details about them in this section.

### 2.2 Titre analysis and purity

Volumetric antibody-peptide fusion titres (Titre) in cell culture supernatants were quantified by protein A affinity chromatography using a protein A ImmunoDetection sensor cartridge (Applied Biosystems, Warrington, United Kingdom) coupled to an Agilent 1,200 series HPLC (Agilent, Berkshire, United Kingdom). Peak areas relative to a reference standard calibration curve were used to calculate titres. These samples were measured on days 8, 10, 12, and 14 for the ambr^®^ 15 system. The trisulfide bond (TSB) was quantified through a TQS triple quadrupole mass spectrometer (Waters, Milford, MA, United States). The monomer purity including fragment and aggregate concentration was monitored with high-performance size exclusion chromatography (HP-SEC) using a TSK-GEL G3000SWXL column (7.8 mm × 30 cm) from Tosoh Bioscience (King of Prussia, PA, United States) with an Agilent 1200 HPLC system (Agilent Technologies, Santa Clara, CA, United States).

### 2.3 Cell line manufacturability index (MI_CL_)

The cell line manufacturability index (*MI*_*CL*_) adapts the standard weighted sum model (Fishburn, 1967) that has been used in the biotech sector to weigh up conflicting criteria for process decisions (Pollock et al., 2017), capacity sourcing decisions (George et al., 2007) and manufacturability indices for formulations (Yang et al., 2017). *MI*_*CL*_ aggregates all available productivity, product quality and growth parameters into a single metric enabling easier evaluation and comparison of *m* cell lines considering *n* criteria:

$$MI_{CL,i}=\sum_{j=1}^{j=n}w_{j}\times r_{ij}\;for\;i=1,2,3\ldots m$$ (1)

where *w*_*i*_ is the normalised weight of each criteria *j* and *r*_*ij*_ is the dimensionless rating of cell line *i* and criteria *j*, calculated as:

$$r_{ij}=\left|\frac{x_{ij}-x_{j,worst}}{x_{j,best}-x_{j,worst}}\right|$$ (2)

where *x*_*ij*_ is the individual ranking of cell line *i* for criteria *j*, *x*_*j,best*_ is the best overall ranking for criteria *j* and *x*_*j,worst*_ is the worst cell line ranking for criteria *j*. *x*_*j,best*_ and *x*_*j,worst*_ are subjectively defined as either a maximum or minimum for each individual criteria based on expert knowledge. For example, for the parameter titre, *x*_*j,best*_ would be the highest titre based on analysing all available titres in the cell line selection and *x*_*j,worst*_ would be the lowest titre concentration. However, for final lactate concentration, *x*_*j,best*_ would be the lowest lactate concentration with *x*_*j,worst*_ equal to the highest concentration. The *w*_*j*_ is the normalised weight of each criteria and can be adjusted for an individual project. The weight can be dependent on several factors including the mode of action of the molecule, market demand, sub-class of molecule (e.g., different weightings and criteria can be selected for monoclonal antibodies (mAbs), fragment antibodies (Fabs), bispecifics, or fusion proteins). The normalised weights ensure that the best possible manufacturability index of an individual cell line *MI*_*CL*_ would be less than or equal to 1, i.e., if a cell line *i* has the best value for each criteria *j* its *MI*_*CL*_ would be equal to 1.

### 2.4 Rule-based natural language generation

The automatically generated report developed in this work implemented a rule-based natural language generation (NLG) algorithm based around the architecture designed for Data-to-Text systems outlined by Becker et al. (2007). The Data-to-Text systems are NLG algorithms that are specifically designed to generate texts from sensor data or other relevant non-linguistic data types. Four stages are required to implement this approach to generate a text from data:

• Signal Analysis: Analysis of the numerical data to identify correlations and useful trends.
• Data Interpretation: Identifying the key messages or patterns within the data.
• Document Planning: Deciding on a document structure that will communicate the key messages from the data analysis and interpretation.
• Microplanning and Realisation: Creating the actual text using the NLG.

Within this work the “Signal Analysis” and “Data Interpretation” stages were completed in steps 2 and 3 of the *CLD_4_* methodology. The “Document Planning” and “Microplanning and Realisation” were completed by Step 4 of the *CLD_4_* and used a simple template to communicate the key messages from the data analysis relevant to the decision making within CLD. The metrics utilised within the report were calculated using Eqs 3, 4. These included the required capacity to meet the production target based on an estimated number of patients and dose size of the drug within the CLD pipeline.

$$D_{product}=N_{patients}\times d_{patient\_year}$$ (3)

$$V_{batch}=\frac{D_{product}}{T_{est}\times N_{batches}\times Y_{DSP}\times P_{s}}$$ (4)

where *D*_*product*_ is the annual product demand (g), *N*_*patients*_ is the estimated number of target patients*, d*_*patient_year*_ is the annual dose required per patient (g)*, V*_*batch*_ is the bioreactor working volume required per batch (L), *T*_*est*_ is the estimated titre from the lead clone (g L^−1^), *N*_*batches*_ is number of batches per year, *Y*_*DSP*_ is the expected overall process yield (%) and *P*_*s*_ is the batch success rate.

### 2.5 Data analysis and visualisation

All online, offline, and meta data were imported and analyzed using algorithms developed in Python 3.9.12 (Python programming language) and Matlab 2021b (The MathWorks, Inc., Natick, MA). R (R Foundation for Statistical Computing, Version 4.1.2, Vienna, Austria) was used to generate the correlation matrix. Matlab 2021b was used to visualise all the graphs.

## 3 Results and discussion

Data is now a major asset for biopharmaceutical companies that continues to grow in both size and complexity, and yet to date this data resource has not yet been fully exploited (Narayanan et al., 2020). This paper helps address this issue within cell line development, where only a small fraction of the available data is utilised in lead clone selection. This work proposes a novel data-driven workflow to improve selection of the lead clone through the analysis of multiple interconnected sources of information using advanced machine learning algorithms. This workflow represents the next-generation of lead clone selection by leveraging all available information for better decision making. The workflow is referenced as *CLD*_*4*_ as it involves four steps and embodies the core principles of Industry 4.0 enabling autonomous data-driven decisions. The first step of *CLD*_*4*_ involves pulling the data in its raw format into a data lake and subsequently classifying the data into different categories, storing the data appropriately within a data warehouse in addition to preprocessing and calculating key features within the data. The second step assesses this structured data within the data warehouse to calculate a new metric referenced as the Cell Line Manufacturability index (*MI*_*CL*_) which ranks all individual cell lines based on selected criteria. The third step involves the application of data analytics using the preprocessed data from step 1 to gain an understanding of how the process operation influences the critical quality attributes and to highlight any potential issues that may occur during scale-up operations and/or commercial manufacturing. Step 4 autonomously generates a report utilising the essential bits of information calculated from Step 1 and the *MI*_*CL*_ from Step 2 to select the lead clone in addition to compiling the key insights generated from the data analytics from Step 3. In addition to selecting the lead clone, this final fourth step provides recommendations supporting long-term business decisions related to capacity planning and future production targets based on additional metadata recorded by the scientists.

### 3.1 CLD_4_ step 1: data classification and storage

The first step of *CLD*_*4*_ was to import all the unstructured raw data related to the cell line development activities into a data lake. A summary of the raw data types is outlined in Table 1. Part of this data transformation involves classifying all available biopharmaceutical data related to CLD activities into five different categories: 1. Process Parameters, 2. Growth, 3. Productivity, 4. Product Quality and 5. Meta-information. A detailed description of the 5 categories used in this work is shown in Table 1 which outlines the frequency, storage system and format of the data.

**TABLE 1:** The five data classifications related to CLD_4_ are summarized by their recording frequency, source and storage format.

Category	Description	Frequency	Source	Format	Examples
Process parameters	Bioreactor process conditions	Every 1–90 s	Single bioreactor system (ambr^®^ 15) includes all data recorded by multiple sensors	Multiple csv files with bioreactor ID and timestamps (proprietary format)	Process data and set-points of dissolved oxygen (*DO*_*2*_), temperature (*T*), pH (*pH*), stirrer speed (*RPM*) and gas flow rates (*F*_*O2*_, *F*_*CO2*_)
Growth	All variables related to cellular growth and nutrient consumption	Every 24 h	Recorded across multiple analytical devices (e.g., YSI 2900, Vi-Cell XR cell counter)	All data saved with timestamp and unique bioreactor reference on internal server and exported as csv/excel format	Glucose (*Gluc*), lactate (*Lact*), viable cell density (*VCD*), growth rate (Spec growth rate)
Productivity	Off-line variables recorded	Every 24/48 h	Recorded across multiple analytical devices (e.g., Agilent 1,200 series HPLC)	Specific to analytical instrument, data exported using Excel based template containing bioreactor ID and timestamp	Titre concentration (*mAb*) and specific productivity (*q*_*antibody*_)

Product quality	Information specific to protein structure	Recorded at harvest of cell culture run	Recorded across multiple analytical devices (SEC-UPLC, Xevo TQS triple quadrupole mass spectrometer)	Specific to analytical instrument, data exported using Excel based template containing bioreactor ID and timestamp	Monomer (*Mono*), aggregation (*Agg*) and trisulfide bond (*TSB*)
Metadata	Non-numerical data recorded, includes cell culture process observations and predicted target market of molecule	Infrequent/not often recorded	No standard recording format, electronic lab notebooks, PowerPoint presentations and management meeting	No standard format, manually recorded by scientist with additional information requested by upper management	Foaming observations, contamination issues, molecule reference, estimated patient dose and estimated market demand

It is evident from Table 1 that one of the major challenges with the consolidation and digitalisation of these raw data sources within the data lake is the multiple formats and storage locations of each data type. This challenge was echoed by Steinwandter et al, (2019) who discussed the multiple data formats and outputs recorded by the different analytical devices and bioreactor systems to be one of the major challenges faced by the biopharmaceutical industry. To help alleviate these issues, there are a number of initiatives such as FAIR data principles (Wilkinson, 2016) that aim to improve infrastructure for better data management and ensure the data is Findable, Accessible, Interoperable and Reusable. Additional standards include the Allotrope Data Format (Millecam et al., 2021) that has the goal of standardising these data formats within industry by ensuring all analytical providers and vendors output their data in a standardised platform-independent data format (i.e., HDF5), which could greatly simplify the consolidation and curation of the data. For this work, an in-house algorithm was developed to gather and store all the data resources shown in Table 1 into a data lake. The raw data then follows an ETL (Extract Transform Load) procedure where the data is extracted from the raw sources and mapped to a large queryable table within a data warehouse. The ETL algorithm imported all the data recorded by the ambr 15 bioreactor that resulted in the importation of a total of 592 csv files (12 csv files per cell culture run and 4 csv files per cell culture station) in addition to importing all other data recorded from each of the analytical devices outlined in Table 2. All this information was converted into a queryable format within the data warehouse based on bioreactor ID, timestamp and source of each data type, this allows all of the online, offline and meta information of each bioreactor run to be easily extracted and used for subsequent analysis. The data was stored as JavaScript Object Notation (JSON) files which is a lightweight format for storing and transporting data, is easy to read and can handle different data types and formats. By structuring this data within a data warehouse, algorithms can be universally applied to the data regardless of previous data format, structure or stage in the screening process which greatly simplifies the application of data analytics. Within this paper the primary focus is on the data recorded during the microbioreactor stage as this stage contains the most amount of information as outlined in Figure 1. Combining data from early and/or late screening could further improve the decision on lead clone provided the cell lines are labelled consistently across the scales and the offline analytics are available for analysis.

**TABLE 2:** A summary of variables and their engineered features within each of the five data categories by *CLD*_*4*_ implementation. Control strategy outlines the objective within the bioreactor to either maximum (Max.), minimise (Min.) or control the variable at its set-point (S.P.).

Variable (Units)		Control strategy	Feature engineering									
			Max		Min	End point	Cumul	Avg	Std	Time above S. P.(hrs)	Time below S.P. (hrs)	
Productivity												
Titre (mg L^−1^)		Max	✓		✗	✓	✗	✓	✗	✗		✗
q_antibody_ (pg cell^−1^ day^−1^)		Max	✓		✓	✓	✗	✓	✗	✗		✗
Growth												
VCD (cells × 10^6^ mL^−1^)		Max	✓		✗	✓	✗	✓	✗	✗		✗
Viability (%)		Max	✓		✓	✓	✗	✓	✗	✗		✗
Lactate (g L^−1^)		Min	✓		✗	✓	✗	✓	✗	✗		✗
Glucose (g L^−1^)		S.P.	✓		✓	✓	✗	✓	✗	✓		✓
Product Quality												
Aggregates (%)		Min	Endpoint only									
Fragments (%)		Min	Endpoint only									
Monomer (%)		Max	Endpoint only									
Trisulfide bond (%)		Min	Endpoint only									
Process Parameters												
Temperature (^o^C)		S.P.	✓		✓	✓	✗	✓	✓	✓		✓
pH (−)		S.P.	✓		✓	✓	✗	✓	✓	✓		✓
DO_2_ (%)		S.P.	✓		✓	✓	✗	✓	✓	✓		✓
Base addition (mL)		S.P.	✗		✗	✗	✓	✗	✗	✗		✗
Flow_O2_ (mL min^-1^)		S.P.	✓		✗	✓	✓	✗	✗	✗		✗
Flow_CO2_ (mL min^-1^)		S.P.	✓		✗	✓	✓	✗	✗	✗		✗
Metadata												
Project ID reference	Scientist	Inoculation date		Bioreactor position		Cell line reference	Projected Titre	Molecule reference		Observations		Estimated patient dose

To simplify the analysis and reduce the complexity of the available time-series data stored in the data warehouse, a selection of features were extracted from this data. The features calculated in this work included the maximum, minimum, end point, cumulative, average, standard deviation, and time above/below set-point as summarised in Table 2. The feature selection method used in this paper is based on industrial experience and the features selected were found to be suitable for this case study. However, Table 2 is not a comprehensive list of features and additional features could be added or removed during on the process. The calculation of these engineered features plays a crucial role in steps 2, 3, and 4 of the *CLD*_*4*_ methodology and has two major advantages. The first is the significant reduction of the data size, this is particularly important for the on-line variables. For example, the pH recorded for an individual ambr^®^ 15 bioreactor cell culture typically contains ∼15,000 data points, based on a 90 s interval for a 16 days period. The majority of the important trends within this pH trend can be summarised by only 6 new features (max, min, end-point, average, std, time above set-point (S.P) and time below S.P) resulting in a drastic reduction of the data in addition to greatly simplifying the subsequent analysis and visualisation of the data. Using the suggested features shown in Table 2, this can be reduced to 6 data points resulting in ∼2,500 fold reduction in data. This data reduction also significantly improves the ease of data analysis in comparison to analysing the complete time-series. Goldrick et al. (2017) highlighted the challenge of using the complete time-series data for end-point predictions of a CQA which involved complex batch-wise unfolding operations, data pre-processing and interpolation methods before a robust PLS and MLR model could be generated.

### 3.2 CLD_4_ step 2: cell line manufacturability index (MI_CL_) calculation

**FIGURE 2:**
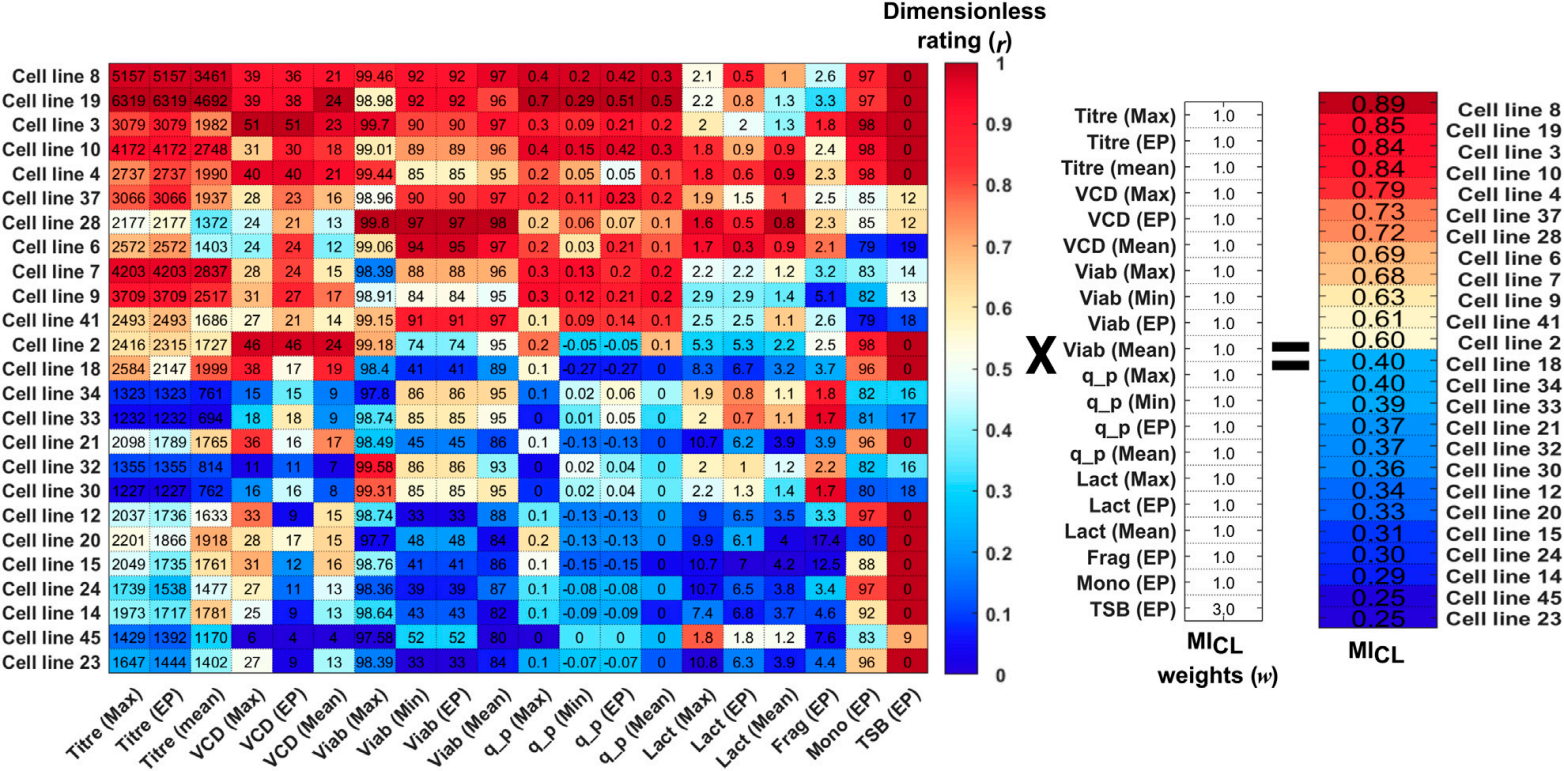
Calculation of the MI_CL_ for the top 12 and bottom 12 clones based on the dimensionless rating (r) of the data from the 20 variables multiplied by the selected weights (w) of each variable. The raw data for each clone is shown in the data matrix and each column is coloured coded from high (dark red) to low (dark blue) based on the variables’ dimensionless rating.

The next step formulates the selection of a lead clone as a multi-criteria decision-making problem enabling scientists to evaluate the performance of each individual clone. The new metric is referenced as the cell line manufacturability index (*MI*_*CL*_). The *MI*_*CL*_ was calculated for the 48 clones using the 20 selection criteria shown in Figure 2. For illustration purposes; Figure 2 shows the top and bottom 12 clones ranked from 1–12 and 36–48 based on their *MI*_*CL*_ value. The *MI*_*C*L_ is a weighted sum metric defined by Eq.1 that considers 20 different selection criteria related to key features extracted in step 1 that summaries the productivity, growth, product quality of each cell line. The rating of each selection criteria was calculated using Eq. 2, where *x*_*i,worst*_ and *x*_*i,best*_, is the worst and best value for each cell line within the run, respectively. The maximum possible value of *MI*_*CL*_ is equal to 1 taking into account the weight of each criteria as defined in Eq. 1. The raw data stored in the data warehouse utilised for the selection of the top clones in this work is shown in Figure 2. Considering the selection criteria for the maximum titre (*Titre (Max)*) of the 48 clones displayed in Figure 2, the lowest (“*worst*”) titre was clone 30 with a titre equal to 1.2 g L^−1^ which equated to a rating value (*r*_*ij*_) of 0, indicated by the dark blue pattern. The highest (“*best*”) titre was cell line 19 with a value equal to 6.3 g L^−1^, resulting in a *r*_*ij*_ of 1, indicated by the dark red pattern. Within the data set evaluated in this study there were three variables which had a reverse rating where the higher the value the lower the rating, i.e., lactate (*Lact*) concentration, aggregate (*Agg*) concentration and trisulfide bond (*TSB*) concentration. For these variables, the lowest experimentally recorded value has the highest rating, i.e., the “best” experimental condition and *vice versa* for the lowest value. This is demonstrated by considering the variable lactate where the lowest (“*best*”) lactate end point (*Lact (EP)*) was 0.3 g L^−1^ produced by Clone 6 and was given a *r*_*i,j*_ rating of 1 and the highest lactate end-point was equal to 7.0 g L^−1^ recorded by clone 15 and given a *r*_*i,j*_ equal to 0.

Each of the selection criteria was given the weights displayed in Figure 2, and for this CLD study, the selection criteria for growth and productivity were all given equal weightings of 1. The choice of equal weighting for growth and productivity was based on scientific experience and knowledge, for example, it has shown previously that high cell densities and low viabilities can lead to a significant increase in process impurities such as lipids, intracellular proteins and nucleic acids (Roush and Lu, 2008). Additionally, it is has been reported that cell lines that remain in a lactate production state and do not switch to a lactate consumption state towards the end of the culture run can yield low cell growth and productivities (Hartley et al., 2018). Therefore, the growth-related parameters of *VCD* (*Max, Mean* & *End-point*), *Viab* (*Max*, *Min* & *End-point*) and *Lact* (*Max, Mean* & *End-point*) were all given equal weighting in comparison to the productivity metrics of Titre (*Max*, *Min*, *Mean*, & *End-point*) and q_p_ (*Max*, *Min &End-point*). The weightings and choice of variables to be included in this analysis can easily be adjusted to account for different user requirement specifications.

However, as a result of the prior knowledge that high TSB concentrations can alter the potency and physical chemical properties of the protein (Goldrick et al., 2017), the TSB concentration at end-point (*TSB EP*) was given a higher weighting equal to 3 to ensure the top clones had minimum TSB levels. Typically, within CLD in addition to the key productivity and growth metrics there may be additional CQAs to be considered to ensure certain quality standards are met. These can include CQAs such as purity, potency, and efficacy which can have an impact on the therapeutic drug’s safety and effectiveness in treating the target disease. Other CQAs can be important for downstream processing such as the concentration of fragments or aggregates. The calculation of the *MI*_*CL*_ can consider multiple CQAs, the only challenge in adding additional CQAs is deciding on their weight as if these CQAs are more important that some CPPs such as final lactate concentration then the weighting may need to be increased. Hence in this case study the TSB concentration was given a weighting of 3 as it was determined to be sufficient to ensure the top 5 clones all had 0% TSB concentrations as can be seen in Figure 2.

**FIGURE 3:**
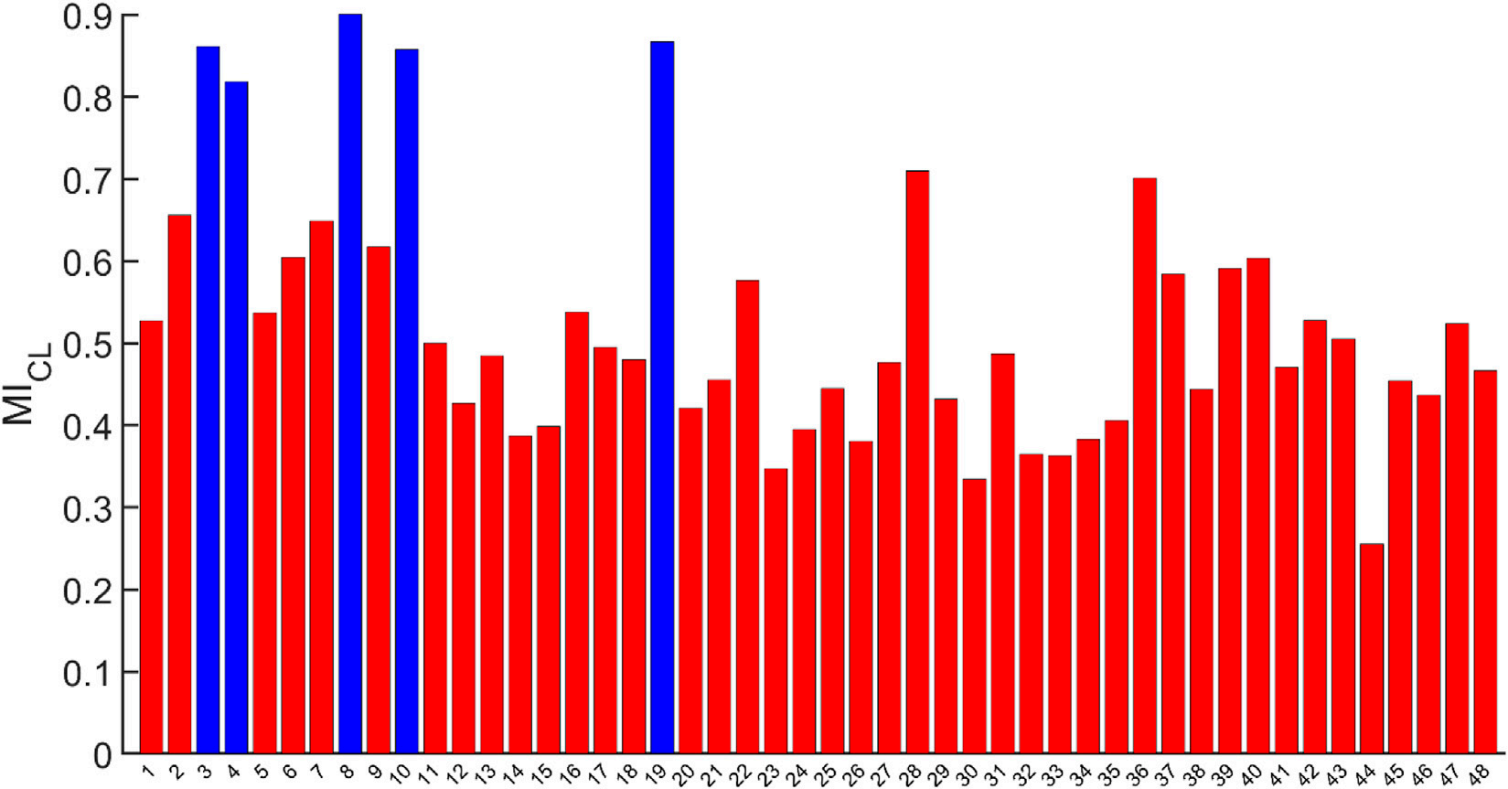
*MI*_*CL*_ calculated for each of the 48 clones of the ambr^®^ 15 that considered all 20 variables with their predefined weighting where the top 5 clones are shown in blue and identified in order as 8, 19, 3 10, and 4.

**FIGURE 4:**
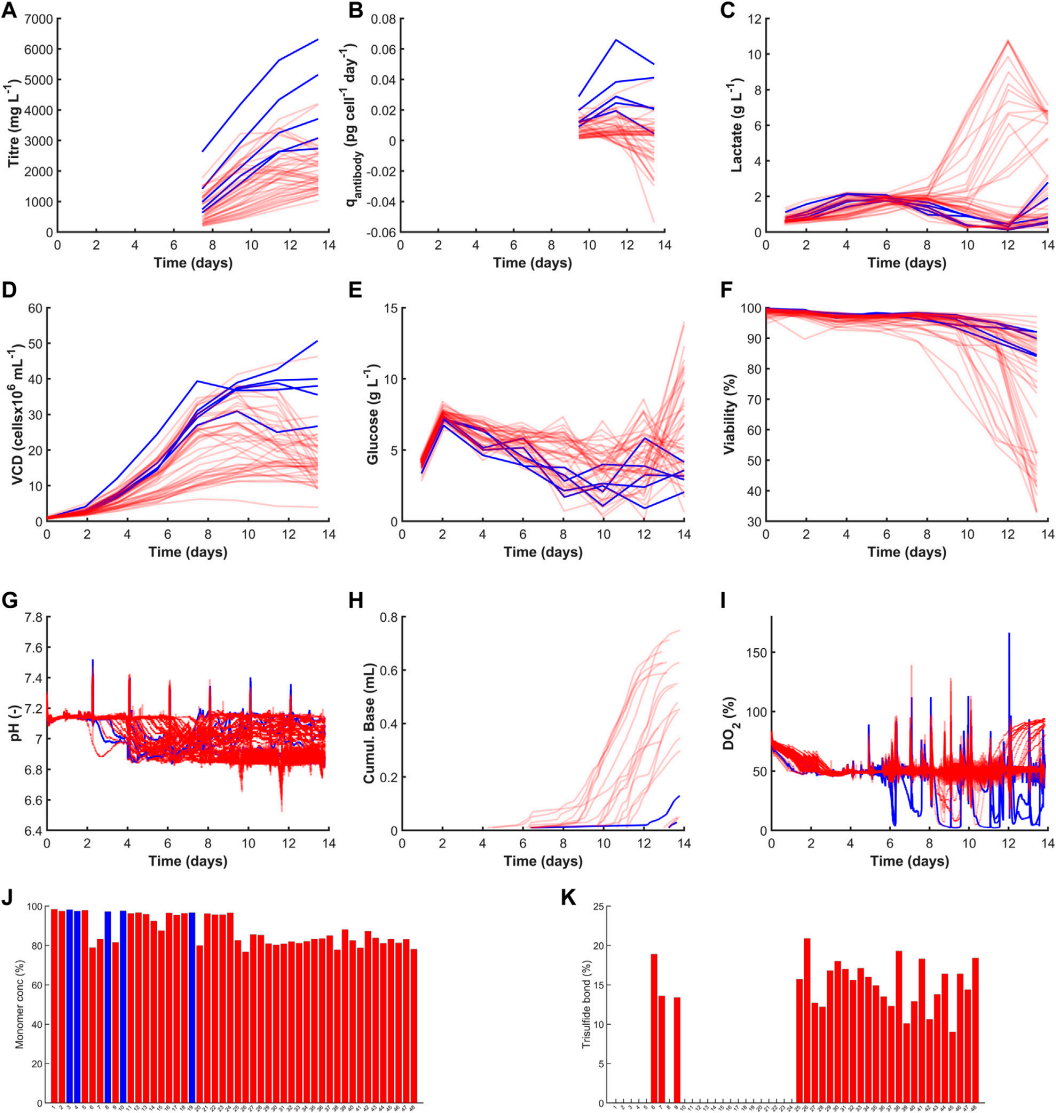
Time course profiles and bar graphs of 48 cell lines considered in this work. The cell lines shown in blue highlight the top cell lines selected using the highest *MI*_*CL*_ values and those in red were not selected. The figures are separated into their four data categories where the productivity graphs are shown in **(A,B)** for Titre (mg L^−1^) and q_Antibody_ (pg cell^−1^ day^−1^). The growth category highlights **(C)** Lactate (g L^−1^), **(D)** VCD (cellsx10^6^ mL^−1^), **(E)** Glucose (g L^−1^) and **(F)** Viability (%). The process parameters are shown in **(G)** pH(−), **(H)** Cumul. Base (mL) and **(I)** DO_2_ (%). The product quality variables are shown by **(J)** Monomer conc (%) and **(K)** Trisulfide bond (%).

Using the selection criteria and weights shown the top five clones were 8, 19, 3, 10, and 4 based on their calculated *MI*_*CL*_ equal to 0.89, 0.85, 0.84 0.84, and 0.79 as shown in Figure 3. To highlight the performance of these top 5 clones in comparison to the other 48 clones considered in this selection process, some variables are shown in Figure 4. The *MI*_*CL*_ provides a simple and comprehensive numerical value summarising the performance of each individual clone considering both the cell lines’ ranking within each criteria and the relative importance of each criteria. This simple metric greatly simplifies the scientist’s decision in selecting the lead clones that demonstrate favourable growth, productivity and product quality values.

Figure 4 highlights time-course and end-point concentrations of selected variables from each of the four categories: productivity, growth, process parameters and product quality. The observed deviations give an early indication of how these clones will respond to the hydrodynamic shear, shifts in cell culture environmental conditions such as pH, temperature and DO_2_ and impact of metabolites concentrations changes such as varying glucose or lactate that may vary at scale-up. The top five clones selected based on the highest *MI*_*CL*_ demonstrated high productivity and favourable growth patterns whilst maintaining desired critical quality attributes. The top two clones yielded high titre concentrations equal to 6.3 g L^−1^ (clone 19) and 5.2 g L^−1^ (clone 5) which is a key criteria to minimise operating costs by reducing the capacity requirements at scale-up and confidently meet market demand (Li et al., 2010; Frye et al., 2016; Priola et al., 2016; Krebs et al., 2018). Clone 19 resulted in a four-fold increase in titre compared to clone 30 with the lowest titre equal to 1.2 g L^−1^. Lin et al. (2019) has investigated the variability in high producing CHO clones and observed a similar 3-4 fold increase from 0.1 g L^−1^ to 0.4 g L^−1^; they attributed the variability to the selection marker and observed the use of attenuated glutamine synthetase (GS) which removed the need for methionine sulfoxamine (MSX) and was shown to generate more stable clones with high productivities. Subsequent process development activities such as adaptive feeding strategies could further increase productivities of these clones as demonstrated by Gagnon et al. (Gagnon et al., 2011), who reported yields in the region of 9–10 g L^−1^. However, in addition to high productivities it is paramount to ensure the product quality remains within specification as highlighted in Figure 4A where clones 7 and 9 outperformed three of the top five clones but as these both had high levels of TSB their overall *MI*_*CL*_ was reduced and therefore they were not selected. Furthermore, this work could be extended to evaluate the risk of the lead clone being out of specification, which may be a useful metric to ensure a stable and robust clone is selected. This demonstrates the flexibility of the *MI*_*CL*_ metric as the weights can easily be changed to select a cell line most appropriate for scale-up and commercialisation.

There were also a significant number of clones that had high lactate metabolism towards the end of a culture run as shown in Figure 4C. The *MI*_*CL*_ takes this into consideration by considering the maximum, end-point and mean lactate concentration; if minimising lactate concentration at the end of the culture is a key criteria then the weight of the end-point lactate (*w*_*LactEP*_) could be increased. This is related to the amount of base added which is significantly lower for the top 5 clones in comparison to the other clones as shown in Figure 4H). The high volume of base added is due to the high number of clones where there was no back metabolism of lactate as shown in Figure 4C), thus additional base is required to maintain the pH at its setpoint. The cell lines with the high base addition and high lactate end-point also had a much lower viability which can also be observed in Figure 4F). The selected five clones were also shown to have high viability with an end-point value of between 88.7% and 92%, this was in stark contrast to some of the other clones that had low viability, e.g., clones 12 and 23 both had an end point viability equal to 33% as shown in Figures 2, 4F. The inclusion of the viability in the *MI*_*CL*_ is paramount as highlighted by researchers that viability below a certain percentage can be problematic to DSP operations (Papathanasiou et al., 2017). The product quality measurements are displayed as bar graphs as only end-points were available for these measurements. Figures 4J, K show the monomer concentration and TSB, respectively with the top 5 clones shown to have the highest purity levels between 96.6% and 98.5% and all had 0% TSB.

### 3.3 CLD_4_ step 3: data analysis

**FIGURE 5:**
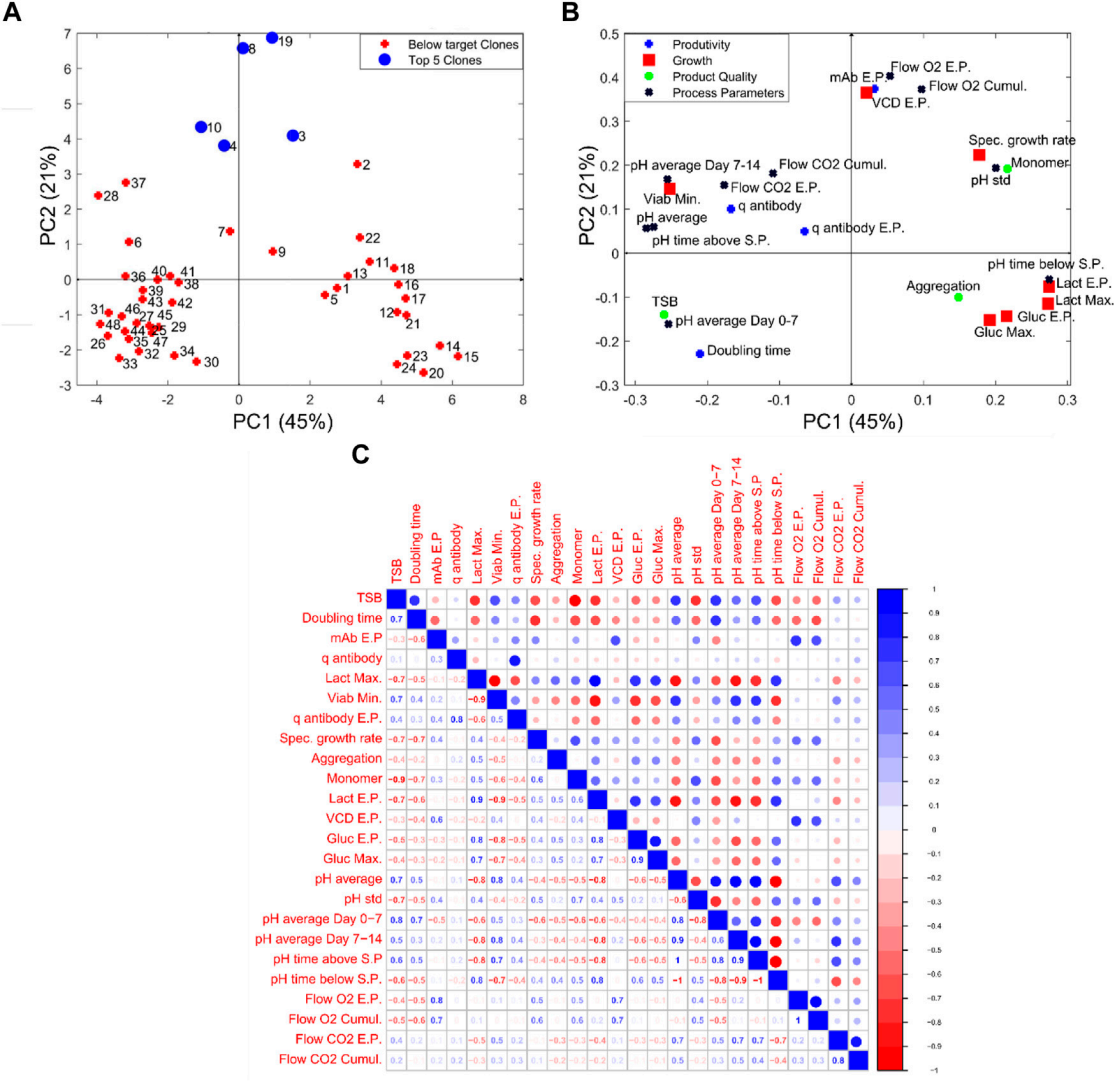
Data analysis of the engineered features for the 48 clones with **(A,B)** showing the scores and loadings graph, respectively, of the first and second principal component (PC) from PCA analysis and **(C)** shows the correlation matrix calculated on the same data.

Steps 1 and 2 of the *CLD*_*4*_ methodology utilise the available data within the categories related to productivity, growth and product quality to select the top 5 clones. Typically, these three categories are the only data types considered during the selection process and the online process parameters defining the cell culture environment are usually ignored. One of the primary reasons for the exclusion of this data is due to the challenge of extracting useful information from these high dimensional data sets. Considering the time-series data shown for the 48 clones shown in Figure 4, it is almost impossible to conclude what is the optimal pH profile (displayed in Figure 4G) or investigate if the dissolved oxygen deviations shown in Figure 4I are influencing the growth parameters. Step 3 of this *CLD*_*4*_ methodology aims to solve these issues by combining feature engineering with the use of ML. The features generated for all the cell culture data was previously discussed in Step 1 of the *CLD*_*4*_. These newly created features were then analysed using principal component analysis (PCA). To negate the impact of different units used by PCA, all of the analysis was done by first normalizing the data by subtracting the mean and dividing by the standard deviation. This normalization process ensures that variables of different units can be evaluated together. The scores and loadings plots of the data analysed are shown in Figures 5A, B, respectively and were generated as described in the materials and methods. The PCA considered a total of 24 features extracted from each of the 48 clones with the first and second principal components accounting for 45% and 21% of the total variance, respectively. The selected parameters used here were based on user experience and could be extended to include more variables. The scores plot shown in Figure 5A classifies the “Top 5 Clones” (Clones 8, 19, 3, 10, and 4) based on achieving the highest *MI*_*CL*_ values (calculated in Step 2) and the remaining clones were classified as “below target clones”. The classification within the loadings plot in Figure 5B is based on the four previously described categories. Through the analysis of the scores and loadings plots, the top five clones cluster together based on similar end-point concentrations of titre (mAb E.P) and VCD (VCD Max) in addition to similar end-point and cumulative flow rates of O_2_ (Flow O2 E.P. and Flow O2 Cumul.). The titres of these clones ranged between 4–6 g L^−1^ and had approximately 40–50 × 10^6^ cells mL^−1^ at harvest as previously shown in Figures 2, 4. The high cell densities associated with these top clones corresponded to the expected high consumption rates of oxygen (Flow O2 EP and Flow O2 Cumul.) as the oxygen uptake rate (OUR) consumption is typically a function of cell density (Deshpande and Heinzle, 2004).

Additional insights can be leveraged by examining the two distinct clusters observed in the PCA figure between the “below target clones” and the “Top 5 clones” with both clusters exhibiting similar process characteristics. The cluster shown in the right quadrants of the scores plot are correlated with high lactate and glucose concentrations (Lact E.P., Lact Max, Gluc E.P., and Gluc Max) and distinguished by their higher pH values (pH time below S.P, pH time above S.P, pH Std). Although each of these clones are controlled using a fixed pH set-point of 7, the wide deadband of the pH controller observed in Figure 4G allows the pH to drift between the upper and lower bands of this pH set-point. These cell culture runs all had high-end point lactate as observed in Figure 4C, where the end-point lactate concentration was as high as 8 g L^−1^. As expected the Pearson’s correlation coefficient (*R* ^*2*^) between the maximum lactate concentrations and minimum viability (Viab min) is equal to 0.9; this reduction in viability was shown to significantly decrease the productivity of these cells and corresponded to negative correlation with productivity values equal to −0.5 as shown in Figure 5C. Similar statistics were reported by Le et al. (2012) through their analysis of 234 cell culture runs and found a *R* ^*2*^ value of −0.87 between final lactate concentration and product yield. This high negative correlation between lactate production and productivity values demonstrates the importance of selecting clones that are shown to back-metabolise lactate but also highlights the influence the environmental conditions within the bioreactor on these clones.

**FIGURE 6:**
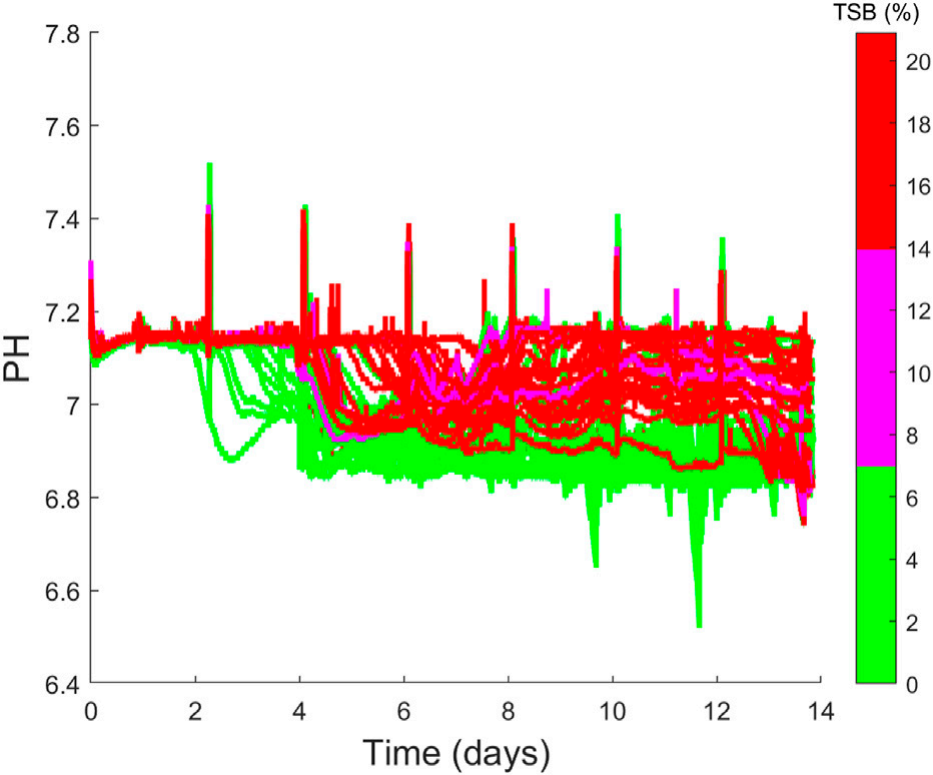
Time series profiles of the pH values of the 48 cell cultures considered which are coloured based on their final TSB (%) concentrations, where low TSB is between 0%–7%, medium TSB is between 7%–14% and high TSB is between 14%–21%.

The second cluster of clones is located to the left of the scores plots and highlights some very interesting correlations between the four cell culture categories of productivity, growth, process parameters and product quality. The cluster primarily consists of clones 36–48 and the most notable variable defining the characteristics of this cluster is the high levels of TSB concentration (10%–20%) associated with each of these clones, which is evident in Figure 4J). The high TSB concentrations were considered an important CQA and therefore gaining an understanding of the process parameters that minimise TSB concentration is of tremendous value for subsequent scale-up activities. Through the analysis of the loadings plot shown in Figure 5B, it suggests high pH operation leads to significantly increased TSB concentrations. This is evident on the loadings graph based on a similar positioning of the TSB and the pH average Day 0–7 & 7–14, pH average, pH time above S.P with respect to PC-1. This is further validated through the correlation matrix shown in Figure 5C, where the TSB concentration and pH average day 0–7 has an *R* ^*2*^ equal to 0.8 and an *R* ^*2*^ equal to 0.7 for both the average pH and pH standard deviation. High pH levels were previously shown to influence TSB concentrations with cell culture operations (Goldrick et al., 2017). The significant influence of pH on the TSB only becomes obvious when the online pH data of the 48 clones is plotted using a coloured based classification of the end-point TSB concentration as shown in Figure 6. Although all the process operation set-points for each clone were fixed some fluctuations around the set-points was observed, this can be seen in Figure 6 where the pH has a set-point equal to 7 with a dead-band equal to 0.1. It is clear from Figure 6 that the clones that operated for the majority of the culture closer to the upper band of the pH dead-band equal to 7.1 had a higher end point concentration of >15% TSB. Whereas, those clones that operated closer to the bottom of the pH dead-band equal to 6.9 had much lower TSB concentrations of between 0% and 6%. It was previously demonstrated that higher pH levels (above 7) towards the end of the cell culture run could promote TSB formation (Goldrick et al., 2017). Additionally it was suggested by (Nielsen et al., 2011) that TSB formation can result from a nucleophilic attack of the sulfide ion (SH^−^) on the disulfide bond of the protein resulting in the formation of a trisulfide bond and this reaction requires a pH at or above neutral, similar to the pH ranges shown for high TSB concentrations shown in Figure 6. Therefore, it is evident that the pH environment of the bioreactor plays a significant role on TSB concentrations and highlights the need for a tighter control of pH to minimise these deviations. During conventional cell line selection screening, the on-line variables such as pH are generally not considered and therefore this issue would not have been identified. However, step 3 of *CLD*_*4*_ allows for these important correlations to be identified and leverages the power of PCA analysis to identify hidden process characteristics that may influence process performance.

### 3.4 CLD_4_ step 4: autonomous report generation

The final step of the *CLD*_*4*_ methodology is to summarise the insights gained within steps 1–3 in an electronic format enabling better decision making to support long-term business objectives and mitigate any potential risks that may impact scale-up activities. Steps 1–3 considered all the information from the four categories of productivity, growth, process parameters and product quality outlined in Table 1. Step 4 takes into account the metadata related to the therapeutic product’s commercial potential that is not fully utilised during the decision making within CLD activities. One of the primary reasons this metadata is not fully harnessed is due to the challenge of extracting valuable and useful information from unstructured text sources. Often important information is communicated through presentations or decisions recorded during a meeting or in an email and is not documented correctly as metadata. Within this work metadata was recorded on target market size for this therapeutic drug in addition to consideration of the available manufacturing capacity within company. The inclusion of this information ensures the required dose and target patient population are considered to ensure the selected lead clone titres can successfully meet production targets given the available manufacturing capacity. Delays in launch of therapeutic drug can be very costly within the biopharmaceutical sector. Therefore, it is imperative that the manufacturing capacity required to meet market demand is considered in this decision making as this may influence the portfolio management of the company and needs to be considered within any budget planning procedures by management (Farid et al., 2020).

**FIGURE 7:**
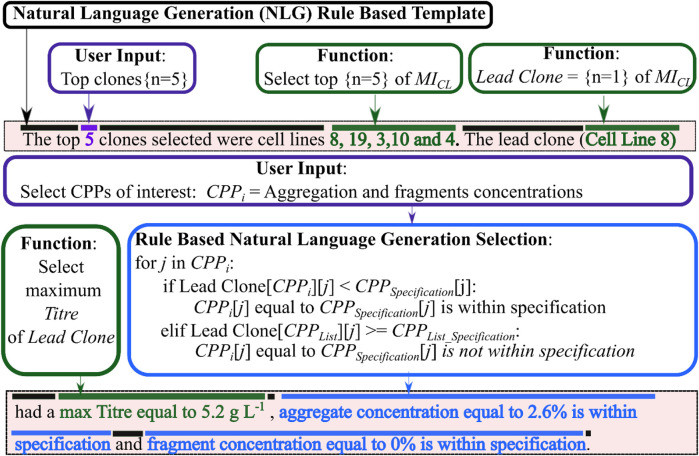
A sample of the natural language generation rule-based template used in this report. The document template text is highlighted in black, user input data highlighted in purple and text generated using a function shown in green.

**FIGURE 8:**
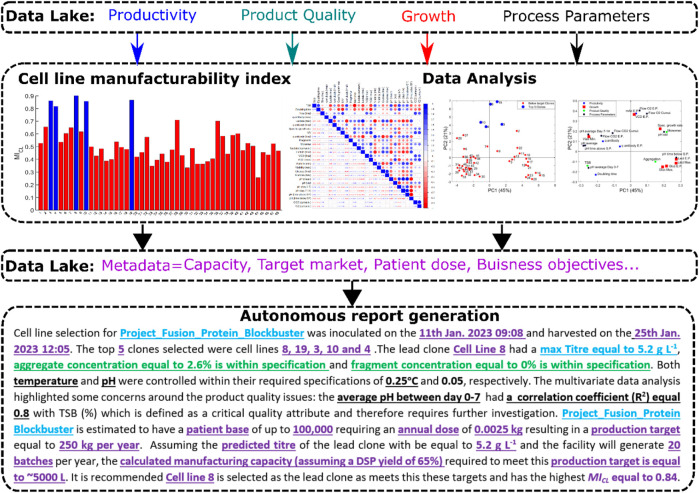
A summary of the *CLD*_*4*_ methodology that includes the Manufacturability Index (MI_CL_), data analysis and NLG report ensuring better lead clones can be selected during CLD that scale-up appropriately and meet the necessary production targets of the drug.

In this work, the final step of *CLD*_*4*_ developed a rule based natural language generation (NLG) algorithm that interpreted all the structured data within the data lake and transformed this information into a human readable report. This report helps contextualise all this information to help the scientists make a more informed data-driven decision when selecting the lead clone. The template-based NLG used a Data-to-Text systems as described in the materials and methods. A sample of the rule-based NLG report is shown in Figure 7, which highlights which text is part of the template, which text requires user input and which text was automatically generated by analysing the data within the data warehouse and using information from steps 1-3 of *CLD*_*4*_. This NLG method was chosen as it is specifically designed to generate texts from sensor data or other relevant non-linguistic data types. Similar reports using this type of Data-to-Text include generating weather reports from weather data (Goldberg, Driedger, and Kittredge, 1994). The rule-based NLG aims to standardise how the results are effectively communicated and to ensure better transparency over this critical decision. NLG algorithms have also been used within healthcare to improve and streamline communication between healthcare professionals and their patients (Cawsey, Webber, and Jones, 1997). Figure 8 includes the final NLG report and highlights the various steps to generate the report. The rule-based NLG is deterministic in nature and ensures consistent and reproducible reports but could be further expanded to utilise more sophisticated algorithms to generate more stochastic reports that may provide more insights from the data analysis.

## 4 Conclusion

The *CLD*_*4*_ methodology presented in this work represents the next-generation process for lead clone selection through advanced data consolidation, analysis and autonomous report generation. Each of the four steps defined by *CLD*_*4*_ leverage value from the high volume of data recorded throughout the CLD operation. The *CLD*_*4*_ evaluated the performance of multiple recombinant CHO cell lines producing high levels of an antibody-peptide fusion with a known product quality issue and recommended selection of the top clone. A key aspect of *CLD*_*4*_ was the creation of a data lake that stored all productivity, process parameter, growth, product quality and metadata recorded by the process in a structured and accessible format. Additionally, the newly created *MI*_*CL*_ metric accessed the performance of 48 clones based on 20 selection criteria. Clone 8 was selected as the lead clone based on its *MI*_*CL*_ value of 0.89 and yielded a product titre of 5.2 g L^−1^ whilst meeting all the required product quality specifications. The subsequent ML identified a strong correlation with the pH of the culture and the end-point concentration of TSB. The correlation indicated cell cultures operating at the higher end of pH dead-band resulted in higher TSB concentrations in the range of 10%–15%. This information provided highly valuable insights and recommendations for the subsequent stability and scale-up studies. The final step of *CLD*_*4*_ automated the generation of a report through a NGL algorithm. This automatically produced report aims to significantly reduce the burden on scientists and engineers through autonomous data analysis in addition to reducing their administrative duties by providing customisable and configurable electronic laboratory notebook entries. Furthermore, this methodology links the laboratory-based activities with the long-term business objectives of the biopharmaceutical company mitigating scale-up risks and ensuring production targets can be met at commercialisation.

## Figures and Tables

**Table:** 

**Agg**	Aggregate
**CHO**	Chinese hamster ovary
**CLD**	Cell line development
**CLD**_**4**_	Cell line development Methodology for Industry 4.0
**CPP**	Critical process parameters
**CQA**	Critical quality attributes
**Csv**	Comma-separated values
**DO**_**2**_	Dissolved oxygen
**DSP**	Downstream processing
**EP**	End point
**ETL**	Extract transform load
**FAbs**	Fragment antibodies
**Gluc**	Glucose
**GS**	Glutamine synthetase
**HT**	High throughput
**HPLC**	High pressure liquid chromatography
**Lact**	Lactate
**mAbs**	Monoclonal antibodies
**MCB**	Master cell bank
***MI***_***CL***_	Cell line manufacturability index
**MSX**	Methionine sulfoxamine
**ML**	Machine learning
**MLR**	Multiple linear regression
**MTP**	Microtitre plates
**NLG**	Natural language generation
**PCA**	Principal component analysis
**PLS**	Partial least squares
**S.P**	Set point
**Std**	Standard deviation
**TSB**	Trisulfide bond
**VCD**	Viable cell density
**Viab**	Viability

## Data Availability

The raw data supporting the conclusion of this article will be made available by the authors, without undue reservation.
